# Demographic and Risk Factor Differences between Children with “One-Time” and “Repeat” Visits to the Emergency Department for Asthma

**DOI:** 10.3390/ijerph18020486

**Published:** 2021-01-09

**Authors:** Pavani Rangachari, Jie Chen, Nishtha Ahuja, Anjeli Patel, Renuka Mehta

**Affiliations:** 1 Department of Interdisciplinary Health Sciences, College of Allied Health Sciences, Augusta University, Augusta, GA 30912, USA; 2 Division of Biostatistics and Data Science, Department of Population Health Sciences, Medical College of Georgia, Augusta University, Augusta, GA 30912, USA; jiechen@augusta.edu; 3 Medical College of Georgia, Augusta University, Augusta, GA 30912, USA; nahuja@augusta.edu (N.A.); apatel17@augusta.edu (A.P.); 4 Division of Critical Care Medicine, Department of Pediatrics, Medical College of Georgia, Augusta University, Augusta, GA 30912, USA; remehta@augusta.edu

**Keywords:** pediatric asthma, emergency department, healthcare utilization, self-management effectiveness, evidence-based practice guidelines, asthma management, “holistic framework”

## Abstract

This retrospective study examines demographic and risk factor differences between children who visited the emergency department (ED) for asthma once (“one-time”) and more than once (“repeat”) over an 18-month period at an academic medical center. The purpose is to contribute to the literature on ED utilization for asthma and provide a foundation for future primary research on self-management effectiveness (SME) of childhood asthma. For the first round of analysis, an 18-month retrospective chart review was conducted on 252 children (0–17 years) who visited the ED for asthma in 2019–2020, to obtain data on demographics, risk factors, and ED visits for each child. Of these, 160 (63%) were “one-time” and 92 (37%) were “repeat” ED patients. Demographic and risk factor differences between “one-time” and “repeat” ED patients were assessed using contingency table and logistic regression analyses. A second round of analysis was conducted on patients in the age-group 8–17 years to match another retrospective asthma study recently completed in the outpatient clinics at the same (study) institution. The first-round analysis indicated that except *age*, none of the individual demographic or risk factors were statistically significant in predicting of “repeat” ED visits. More unequivocally, the second-round analysis revealed that none of the individual factors examined (including *age*, *race*, *gender*, *insurance*, and *asthma severity*, among others) were statistically significant in predicting “repeat” ED visits for childhood asthma. A key implication of the results therefore is that something other than the factors examined is driving “repeat” ED visits in children with asthma. In addition to contributing to the ED utilization literature, the results serve to corroborate findings from the recent outpatient study and bolster the impetus for future primary research on SME of childhood asthma.

## 1. Introduction

Asthma is the most common pediatric chronic disease in the US, affecting nearly 10 million children (approximately 15%) under 18 years of age [1,2,3]. It is also among the top three leading causes of hospitalizations among children. Acute asthma exacerbations require the patient to seek immediate (unscheduled) healthcare, including emergency department (ED) visits and hospitalizations. Nearly 5 million children experience an asthma exacerbation each year in the US, accounting for an estimated 15 million missed school days, nearly 2 million ED visits, and greater than 60% of asthma-related costs. Past studies evaluating recurring visits (within one year) to pediatric EDs have found asthma to be the most common diagnosis within all recurring-visit groups and the most common diagnosis for high-frequency users (four or more recurring visits). In 2010, an evaluation of state Medicaid programs estimated a combined USD 272 million spent on asthma-related ED visits among children [1,2].

This study seeks to examine demographic and risk factor differences between children who visit the ED for asthma once (“one-time” ED patients) and those who visit the ED more than once (“repeat” ED patients) over an 18-month retrospective period, at an academic medical center. Before delving into the methods and results of this study, it would be important to take note of some essential background information for this study (described in the next section), which serves to provide not only the rationale for conducting this study, but also the broader context for interpreting its results.

## 2. Background

### 2.1. Pediatric Asthma-Related ED Visits Can Be Prevented by Improving Self-Management Effectiveness (SME)

According to the 2007 National Heart Lung and Blood Institute (NHLBI) evidence-based guidelines for asthma management and recent meta-reviews of the literature, “unscheduled healthcare use” for asthma, including ED visits, hospitalizations, and urgent care visits, can be prevented through effective self-management of asthma [4,5,6,7,8]. To this effect, the guidelines emphasize the importance of creating a provider–patient partnership to activate patients to regularly monitor asthma symptoms, make necessary treatment adjustments, and take control of their disease. Concurrently, recent meta-reviews of studies on “supported self-management of asthma” have determined that interventions targeting the combination of *patient*, *provider*, and *organizational* factors demonstrated the greatest improvement in health outcomes, compared to targeting patients or providers alone. As such, both the NHLBI guidelines and recent empirical research have underscored the need for increasing provider and organizational (hospital/clinic) engagement in asthma management. Nevertheless, gaps in adherence to NHLBI guidelines in routine clinical practice continue to persist.

### 2.2. Improving Pediatric Asthma SME Requires a “Holistic Framework” for Measuring SME

For widespread, systematic provider engagement in improving asthma SME (in routine clinical practice), a comprehensive set of resources for measuring SME is necessary, which currently does not exist. This gap could be attributed the fragmented nature of the asthma management literature. To date, no study has examined the concurrent impact of “provider–patient/family communication” and “patient/family activation” on both “medication adherence” (intermediate outcome of SME) and “unscheduled healthcare use” (primary outcome of SME) for asthma, while also controlling for an array of socio-ecological influences on asthma SME. Additionally, the existing literature on asthma SME has been largely restricted to cross-sectional studies, with limited use of validated measures of the aforementioned key constructs. Recognizing these gaps in the literature, researchers have recently worked to develop a “holistic framework” for measuring SME in childhood asthma [9,10]. The rationale behind this framework is as follows:

- Extrinsic socioecological factors (individual demographic and risk factors, interpersonal factors, socioeconomic factors, health system factors including provider–patient/family communication on asthma management, community-level factors, and environmental-level factors) can impact
- Intrinsic self-agency factors (patient/family activation in asthma management), to ultimately influence
- SME in childhood asthma. SME in turn is defined by both primary outcomes (unscheduled healthcare use in children with asthma) and intermediate outcomes (asthma medication adherence and symptom control).

A figure summarizing the “holistic framework” for measuring SME in childhood asthma is available in an earlier (open access) publication led by the lead author [10]. The purpose of the “holistic framework” is to guide empirical research investigating a comprehensive set of factors influencing childhood asthma SME and their interrelationships, to create a robust evidence base for generating resources for providers to measure and improve childhood asthma SME.

### 2.3. Need for Incremental Retrospective Studies to Develop Rationale for a Holistic Primary Study to Measure and Improve SME of Pediatric Asthma

Since a full-fledged research study informed by the “holistic framework” would involve substantial primary data collection efforts, an incremental approach to investigating interrelationships among key variables in the framework through retrospective chart review efforts could serve a dual purpose in (**1**) providing insight into key variables influencing SME while also (**2**) providing the foundation and rationale for a full-fledged study.

In keeping with this rationale, a first step to investigating interrelationships among variables in the “holistic framework” through retrospective chart review was undertaken in a recent study of demographic and risk factor differences between users and non-users of unscheduled healthcare for childhood persistent asthma. This earlier study was set in the outpatient clinics at the same institution and took a first step towards assessing SME in 59 children with asthma (8–17 years), informed by the “holistic framework”, by defining SME in terms of the primary outcome of unscheduled healthcare use. In the outpatient study, “users” of unscheduled healthcare served to represent “low SME”, while “non-users” of unscheduled healthcare within the same asthma severity category served to represent “high SME” [11]. The study examined differences between users and non-users of unscheduled healthcare in persistent childhood asthma, with respect to select individual demographic and risk factors obtained through record review, including *asthma severity* (defined as mild-persistent, moderate-persistent, and severe-persistent asthma), *age, gender, race, insurance*, and *body-mass index (BMI).*

The results of the outpatient study were eye-opening. All three persistent asthma severity categories contained users and non-users of unscheduled healthcare, with “moderate-persistent” having a roughly equal distribution of users (11; 44%) and non-users (14; 56%). “Mild-persistent” had 4 users and 16 non-users, while “severe-persistent” had 10 users and 4 non-users. The only statistically significant finding from the study was that the “mild-persistent” category had fewer users than the “severe-persistent” category. However, after adjusting for severity, there were no significant differences between users and non-users on any other factor examined. In other words, although severity was statistically significant in explaining higher use of unscheduled healthcare, the fact remained that there were users and non-users of unscheduled healthcare use in all three severity categories for persistent childhood asthma. After adjusting for severity, none of the individual demographic and risk factors were statistically significant in explaining use of unscheduled healthcare in persistent childhood asthma. A key implication of these findings is that something other than the individual factors examined is driving unscheduled healthcare use in children with persistent asthma. Results from the outpatient study provided impetus for future primary research on factors influencing the SME of childhood asthma informed by the “holistic framework” including the roles of specific components of supported self-management of asthma, such as “provider–patient/family communication” and “patient/family activation”, in explaining differences in unscheduled healthcare use in childhood asthma.

The retrospective study described in this paper is set in the ED at the same (study) institution as the outpatient study. This study seeks to serve as a reinforcing incremental study seeking to understand factors influencing the SME of childhood asthma through retrospective review in order to provide impetus for future primary research on measuring and improving childhood asthma SME, informed by the “holistic framework”. Although the outpatient study served as an initial retrospective study, it was limited in sample size and restricted to a single setting. Obtaining results from a larger sample size in a different (ED) setting that serve to corroborate results from the outpatient setting has the potential to strengthen the rationale and foundation for future primary research. To this effect, the primary outcome of ED visits for childhood asthma in this study, which, in turn, is a prime example of unscheduled healthcare use, will serve as a proxy for SME of childhood asthma, as informed by the “holistic framework”. The remaining portion of this paper describes the purpose, methods, and results of this study.

## 3. Purpose

The research question of interest to this study is: What are the demographic and risk factor differences between children with “one-time” and “repeat” ED visits for childhood asthma? As described above, a key purpose of this paper is to provide a foundation for future research on childhood asthma SME, informed by the “holistic framework”. However, the paper also seeks to serve an additional purpose of contributing to the literature on ED utilization for childhood asthma. The existing literature has found the biggest determinants of ED utilization for childhood asthma to be access to care, disease severity, and socioeconomic factors. However, the vast majority of these studies have been undertaken among uninsured children and children with insurance coverage gaps [1,2,3,12,13,14]. The population of interest to this study is an insured population with access to primary and specialty outpatient care for childhood asthma. An understanding of demographic and risk factors influencing ED use for childhood asthma in this population therefore would serve as a useful complement to the existing literature.

In summary, the objective of this study is to examine differences between children who visit the ED for asthma once (“one-time” ED patients) and those who visit the ED more than once (“repeat” ED patients) over an 18-month retrospective period with respect to various demographic characteristics and risk factors. By gaining a better understanding of the individual factors influencing ED use for childhood asthma, this study seeks to serve the dual purpose of:

(1) Providing a foundation for future primary research on measuring and improving childhood asthma SME, informed by the “holistic framework”;
(2) Contributing to the literature on ED utilization for childhood asthma.

## 4. Methodology

The setting for this retrospective study was the emergency department (ED) at a children’s medical center, located within a public tertiary academic health system in Augusta, Georgia, USA. The population of interest was all unique pediatric patients (0–17 years) who visited the ED for primary clinical diagnosis of asthma between 1 January 2019 and 30 June 2020. Children were included in the study if they belonged to any of the following four severity categories for asthma (defined by NHLBI guidelines): intermittent, mild-persistent, moderate-persistent, or severe-persistent. Children with the potential for unrelated respiratory disease, including those with cystic fibrosis, congenital cardiac comorbidities, respiratory disease of prematurity, primary immunodeficiency, and neuromuscular disorders, were excluded from the study. The chart review was conducted by two medical students, under the supervision of a pediatric critical care faculty physician.

For the children who met study eligibility criteria, data were collected in the following three categories of data elements informed by the “holistic framework” (discussed earlier), through an 18-month retrospective review of each individual medical record:

- *Individual demographic factors*, including: Round 1 analysis among 0–17-year-old children (<5 years, 5–9 years, >9 years); Round 2 analysis among 8–17-year-old children (8–12 years, 13–17 years); gender (male or female); race (Caucasian, African-American, other); insurance (Medicaid, private, other).
- *Individual risk factors*, including: asthma severity category (intermittent, mild-persistent, moderate-persistent, or severe-persistent); BMI, defined as normal (<85%), overweight (85–95%), or obese (>95%); encounter type (ED-only; ED-to-inpatient; transfer ED-to-inpatient); primary care physician (PCP) (within study institution; outside study institution); encounter length of stay (LOS) (0 days; 1 day; 2 days; >2 days); socioeconomic risk (low, moderate, high).
- *Primary outcome of childhood asthma SME* was assessed based on the number of ED visits over an 18-month retrospective timeframe and defined as: (1) patients who visited the ED once over the previous 18 months (“one-time”); (2) patients who visited the ED more than once over the previous 18 months (“repeat”).

While most of the data elements listed above are straightforward, a couple of them require additional clarification. For example, it would be relevant to note that the variable “encounter type” excludes all direct inpatient admits to the hospital (i.e., admissions that do not pass through the ED); the variable “socioeconomic risk” was obtained by aggregating five categories of data from record review: (**1**) housing or transportation issues, defined by notes by caregivers or social workers about mode of transport or change of addresses; (**2**) smoking exposure, defined as any exposure within the immediate family; (**3**) previous unplanned admission at any time, which would be indicated by a social work note; (**4**) more than three ED visits at any time, at any location, which would be indicated by a social work note; and (**5**) social dynamic factors, including any indication of familial or custody issues that could negatively affect the patient’s care. Based on the aggregated values, “socioeconomic risk” was categorized as “high”, “moderate”, or “low.”

There were two reasons for performing the second-round analysis for the age range 8–17 years. Firstly, this age interval is typically used for pediatric studies involving primary data collection, since children ≥ 8 years can be more confidently approached for completion of surveys and interviews (with parental consent) compared to children < 8 years [15,16]. Since future primary research on measuring and improving childhood asthma SME is being planned, it would be important for the preliminary retrospective studies to mirror those age ranges to enhance the comparability of the results. Secondly, there is a clinical rationale for preferring ≥ 8 years of age for studies related to pediatric asthma, since clinical diagnoses of asthma, including chronic, persistent asthma can be more reliably established in children ≥ 8 years of age due to the ability to perform pulmonary function testing (PFT), which is not feasible to implement for lower age-groups [17,18].

Differences between children with “one-time” vs. “repeat” visits to the ED were assessed with respect to aforementioned individual demographic and risk factors, using contingency table and logistic regression analysis.

## 5. Results

In Round 1 analysis (0–17 years), there were a total of 252 unique patients (children) who visited the ED at the study institution for asthma between January 2019 and June 2020. A retrospective review of each individual record over an 18-month timeframe revealed that 160 (63%) were “one-time ED” patients, whereas 92 (37%) were “repeat ED” patients. In other words, 92 patients visited the ED more than once over an 18-month timeframe. It would be relevant to note that although a total of 252 individual records were reviewed, not all contained complete data on every individual demographic and risk factor variable examined. Some of these variables contained missing values, as summarized in Table 1 for Round 1 analysis. To clarify, the chart review process, which was conducted by clinical experts (two medical students under the supervision of a pediatric critical care physician), did in fact utilize a standardized data collection sheet. However, the process of note-writing at the study institution can vary by provider/clinician. As such, there were instances of missing information owing to a lack of medical record documentation (which was not in the control of the chart reviewers).

In Round 2 analysis (8–17 years), there were a total of 139 unique patients (children) who visited the ED at the study institution for asthma between January 2019 and June 2020. A retrospective review of each individual record over an 18-month timeframe revealed that 96 (69%) were “one-time ED” patients, whereas 43 (31%) were “repeat ED” patients. It would be relevant to note that although a total of 139 individual records were reviewed, not all contained complete data on every individual demographic and risk factor variable examined. Some of these variables contained missing values, as summarized in Table 2 for Round 2 analysis.

Contingency table analysis and logistic regression analysis were conducted in both Round 1 and 2. Both rounds of logistic regression analysis included seven key independent variables (IVs): *asthma severity*, *age*, *gender*, *race*, *insurance*, *BMI*, and *socioeconomic risk*. Excluding “encounter length of stay” and “encounter type” from the regression model was appropriate, since both these variables could serve as proxies for “asthma severity”. Importantly, restricting the logistic model to seven IVs in both rounds of analysis enabled comparability of the results between rounds and with the earlier (aforementioned) outpatient study, while also ensuring adherence to the statistical convention of 10-plus observations for every included IV in logistic regression, for valid statistical results [19]. Round 1 results from the contingency table and logistic regression analysis are presented in Table 3 and Table 4, respectively, while Round 2 results from the contingency table and logistic regression analysis are presented in Table 5 and Table 6, respectively.

### 5.1. Round 1 Results

**Figure 1:**
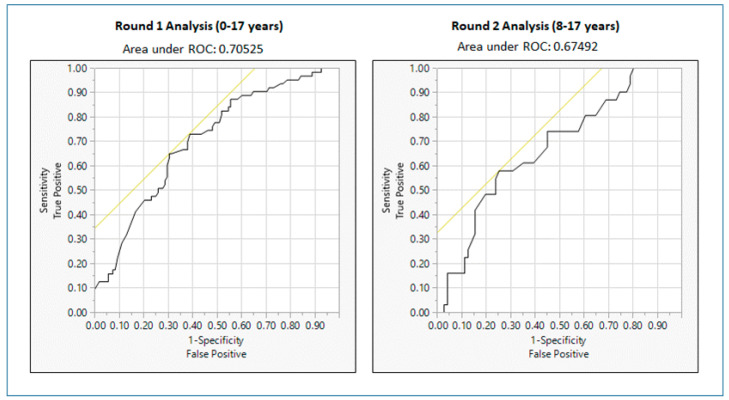
Receiver Operating Characteristic (ROC) curves for Round 1 and Round 2 analysis.

Results from Round 1 analysis revealed that although a few variables, including *age*, *insurance*, and *socioeconomic risk* emerged as statistically significant in the contingency table analysis (Table 3), only *age* was statistically significant in the logistic regression model, after adjusting for all other variables. The Chi-squared goodness-of-fit test was 25.94068 (df = 14) with *p*-value = 0.0263 indicating that the overall logistic regression model is significant. In addition, the area under the Receiver Operating Characteristic (ROC) curve was 0.7053 (greater than 0.50), confirming that the logistic regression model is adequate for this analysis. Figure 1 shows the ROC curves corresponding to logistic regression models for Round 1 and Round 2 analyses. As indicated in Table 4 (in the odds ratio section), children 5–9 years of age, were three times more likely than children under <5 years of age to have “repeat” ED visits (i.e., visit the ED more than once over an 18-month period). Other than *age*, no variable, including *asthma severity*, was significant in predicting “repeat” visits to the ED.

### 5.2. Round 2 Results

Results from Round 2 analysis were unequivocal in indicating that none of the individual demographic or risk factors examined (not a single one) were significant in predicting “repeat” visits to the ED for childhood asthma, neither in the contingency table analysis (Table 5) nor in the logistic regression analysis (Table 6). The Chi-squared goodness-of-fit test was 9.160775 (df = 11) with *p*-value = 0.6071, indicating that the overall logistic regression model is not significant. However, the area under the ROC curve is 0.6749 (greater than 0.50), confirming that although the logistic regression model is not significant in this case due to smaller sample size, it is acceptable for this analysis.

## 6. Discussions

To summarize the results, in Round 1 analysis (0–17 years), except for age, no other individual demographic or risk factor was significant in predicting “repeat” ED visits for childhood asthma. Results showed significantly higher likelihood of “repeat” ED visits in the 5–9 years age-group compared to the <5 years age-group. This could be owing to the fact that children 5–9 years may be more able to articulate their symptoms and concerns to their parents, prompting more ED visits. However, as discussed under Methodology, the potential limitations to the reliability of asthma clinical diagnosis in ages below 8 years (and particularly below 5 years) need to be acknowledged while interpreting this result. The latter concern in turn makes Round 2 results (8–17 years) particularly relevant for verifying Round 1 results and consolidating our understanding of the role of the individual demographic and risk factors examined in predicting “repeat” ED visits. Round 2 results are particularly relevant when the above concern is considered in conjunction with the fact that the 8–17 years age interval has been preferred in primary pediatric research, owing to higher confidence in the ability of children 8 years and older to reliably complete surveys and interviews related to asthma management (with parental consent) [15,16]. Importantly, since Round 2 results are based on children ages 8–17 years, they are directly comparable to results from the outpatient study at the same institution. As indicated in Table 5 and Table 6, Round 2 results were unequivocal in revealing that not a single one of the individual demographic or risk factors examined was statistically significant in predicting “repeat” ED visits for childhood asthma (neither in contingency table analysis nor the logistic regression analysis).

The outpatient study (described under Background) examined the same age-group as Round 2 of this (ED) study and found that other than *asthma severity*, no other individual demographic and risk factor examined was significant in predicting unscheduled healthcare use (including ED visits) for asthma. Moreover, *asthma severity* was only found to be significant in explaining the number of users of unscheduled healthcare in the “mild-persistent” category compared to “severe-persistent”. The fact remained that there were users and non-users of unscheduled healthcare in all three persistent asthma severity categories (mild, moderate, and severe) and none of the individual demographic or risk factors examined served to explain differences in the use of unscheduled healthcare across the three severity groups [11]. A key implication therefore was that something other than the individual factors examined was driving unscheduled healthcare use. These findings in turn served to provide impetus for future research on drivers of unscheduled healthcare use and SME for childhood asthma informed by the “holistic framework”. Round 2 results (for 8–17 years) not only serve to corroborate the results of the outpatient study, but they go a step further in finding that even *asthma severity* (defined as intermittent, mild-persistent, moderate-persistent, and severe-persistent asthma) was not significant in predicting “repeat” ED visits for childhood asthma, a result that was also indicated in Round 1 analysis. These findings suggest that the results from this (ED) study are equally, if not more emphatic (than the outpatient study), in providing impetus for future research on understanding the drivers of unscheduled healthcare use and SME of childhood asthma, informed by the “holistic framework”.

Importantly, Round 2 results serve to contribute to the literature on ED utilization for asthma. Although the existing literature has found the biggest determinants of ED utilization for childhood asthma to be access to care, disease severity, and socioeconomic factors, the vast majority of these studies have been undertaken among uninsured children and children with insurance coverage gaps [12,13,14]. The pediatric asthma patient population in this study however is largely insured, with access to primary care for asthma. None of the patients were uninsured. It would also be relevant to note that the existing literature on ED utilization in the insured population has found race and severity to be significant predictors of recurrent ED visits [1,2,3]. This study did not find *asthma severity* and other socioeconomic factors like *race* and *insurance* to be significant predictors of “repeat” ED visits, suggesting that future research may be needed to understand key drivers of “repeat” ED visits and unscheduled healthcare use for childhood asthma in a largely insured population with access to primary and specialty care. In summary, congruent with its intended purpose, this study serves the dual purpose of (**1**) providing a foundation for future primary research on measuring and improving childhood asthma SME, and (**2**) contributing to the literature on ED utilization for childhood asthma.

### 6.1. Study Limitations and Strengths

Limitations of this study include reliance on retrospective chart review, which restricts data availability to data contained in the medical record. It would also be relevant to note that there was limited variability in race in the study population, with 87% being African-American. It would be relevant to note however that the outpatient study population (at the same institution) had greater variability in race, with 69% being African-American. In the outpatient study, race was not found to be a significant predictor of unscheduled healthcare use (including ED visits), and in this (ED) study, race was not found to be a significant predictor of repeat ED visits for childhood asthma. Another limitation of this study was missing data for some variables. It would be relevant to note however that despite this limitation, we were left with a healthy sample size of *n* = 171 for Round 1 logistic regression and *n* = 102 for Round 2 logistic regression. It must also be acknowledged that patient visits to other EDs in the community may have been missed in the absence of documentation; however, this is less of a concern given that the study is set in a large academic health system providing primary, secondary, and tertiary care, with most of the children (patients of the health system) known to receive all their care within the institution, including emergency visits and hospitalizations.

Key strengths of the study include the use of clinical experts (two medical students and supervising physician for chart review) and an adequate sample size of *n* = 252 for Round 1 analysis and *n* = 139 for Round 2 analysis, both of which significantly exceed the statistical rule-of-thumb of *n* = 30 for statistical significance. The study location may also be viewed as a strength. Augusta, Georgia is located in an area of the southern U.S. that has elevated rates of morbidity and mortality from a variety of chronic diseases, especially asthma. In 2015, Augusta was featured in the “Top Ten Asthma Capitals of the US” by the Asthma and Allergy Foundation of America [20]. Our sample of asthma-vulnerable children therefore may be representative of other high-risk rural or inner-city U.S. outpatient settings for asthma treatment. Moreover, the higher asthma severity patient base served by the medical center as a whole is highly relevant to understanding unscheduled healthcare use in child asthma, as echoed by the Pareto Principle on healthcare use: 80% of unscheduled (costly) healthcare use could be attributed to the 20% severely-ill populace [21].

### 6.2. Implications for Future Research and Practice

The study provides important implications for future research and practice in childhood asthma management. In both the outpatient study and this ED study, the differences in use of unscheduled healthcare for childhood asthma (including repeat ED visits in this study) were not explained by any of the individual demographic and risk factors examined (after adjusting for *asthma severity*). A key implication of these results therefore is that other factors (not examined) are at play in driving “repeat” ED visits and unscheduled healthcare use (“low SME”) for childhood asthma. These findings in turn provide the impetus for future primary research on measuring and improving SME of childhood asthma, informed by a “holistic framework”.

Although the NHLBI guidelines have emphasized the importance of provider–patient partnership in improving asthma SME, and systematic reviews have determined that “supported self-management of asthma” can reduce unscheduled healthcare use across a wide variety of populations, the evidence pertaining to the interrelationships among provider–patient/family communication, patient/family activation, and asthma SME remains fragmented. For example, a 2017 study examining the relationship between physician–patient communication and self-care skills for asthma was limited by: (**1**) defining self-care skills solely in terms of asthma medication adherence (as opposed to medication adherence, environmental trigger avoidance, and symptom control as a whole); (**2**) not considering any health outcome measures, including healthcare use for asthma; (**3**) not utilizing validated measures of physician–patient communication; (**4**) not accounting for the impact of a variety of socio-ecological influences on self-care skills; and (**5**) relying entirely on a retrospective cross-sectional study design [22].

The fragmented nature of the asthma SME literature in turn mitigates understanding of the specific mechanisms by which “supported self-management of asthma” reduces unscheduled healthcare and strengthens SME, which in turn serves as a barrier to: (**i**) systematically engaging providers in measuring and improving asthma SME; (**ii**) identifying holistic interventions for improving and sustaining asthma SME (for future evaluation); and (**iii**) ensuring consistent implementation of NHLBI guidelines in routine clinical practice.

A full-fledged primary research study for measuring and improving SME of childhood asthma (informed by the “holistic framework”) has the potential to address these gaps in the literature. This type of comprehensive study of factors influencing childhood asthma SME (and unscheduled healthcare use) in turn has the potential to generate resources and tools for asthma providers to measure SME in the clinic setting, and improve SME in real-time, through effective, targeted interventions, in line with NHLBI practice guidelines. In summary, future studies informed by the “holistic framework” have the potential to serve the dual purpose of addressing gaps in the asthma management literature, while tackling the practical challenges of childhood asthma management.

## 7. Conclusions

This study examined demographic and risk factor differences between children who visited the ED for asthma once (“one-time”) and more than once (“repeat”) over an 18-month retrospective timeframe at an academic medical center. The essential finding of the study is that none of the individual risk factors examined, including *age* (among 8–17 year olds), *race*, *gender*, *insurance*, and *asthma severity*, were statistically significant in predicting “repeat” ED visits for childhood asthma. A key implication therefore is that something other than the individual demographic and risk factors examined is driving “repeat” ED visits in children with asthma. The results serve to contribute to the asthma-related ED utilization literature. Importantly, they serve to complement findings from a recent outpatient study (at the same institution) and bolster the impetus for future primary research on measuring and improving SME of childhood asthma. In the longer term, such future research has the potential to alleviate the economic and public health burden of asthma, with implications for managing other chronic diseases.

## Figures and Tables

**Table 1:** Summary data on study variables: Round 1 analysis (0–17 years).

	**N**	**%**
***ED Visit***		
One-Time ED Patient	160	63%
Repeat ED Patient	92	37%
**Total**	**252**	**100%**
***Individual Patient Demographics***		
Age: <5 years	38	15%
Age: 5–9 years	119	47%
Age: >9 years	95	38%
Age: Missing Values	0	0%
**Total**	**252**	**100%**
Gender: Male	168	67%
Gender: Female	84	33%
Gender: Missing Values	0	0%
**Total**	**252**	**100%**
Race: Caucasian	23	9%
Race: African American	212	84%
Race: Other	16	6%
Race: Missing Values	1	0%
**Total**	**252**	**100%**
Insurance: Medicaid	182	72%
Insurance: Private	58	23%
Insurance: Other	9	4%
Insurance: Missing Values	3	1%
**Total**	**252**	**100%**
***Individual Patient Risk Factors***		
Asthma Severity: Intermittent	41	16%
Asthma Severity: Mild-Persistent	81	32%
Asthma Severity: Moderate-Persistent	58	23%
Asthma Severity: Severe-Persistent	8	3%
Asthma Severity: Missing Values	64	25%
**Total**	**252**	**100%**
BMI: Normal	132	52%
BMI: Overweight	46	18%
BMI: Obese	42	17%
BMI: Missing Values	32	13%
**Total**	**252**	**100%**
Encounter Type: ED-to-inpatient	91	36%
Encounter Type: Transfer ED-to-inpatient	4	2%
Encounter Type: ED-only	157	62%
Encounter Type: Missing Values	0	0%
**Total**	**252**	**100%**
Encounter LOS: 0 days	135	54%
Encounter LOS: 1 day	78	31%
Encounter LOS: 2 days	23	9%
Encounter LOS: >2 days	15	6%
Encounter LOS: Missing Values	1	0%
**Total**	**252**	**100%**
PCP: Within Study Institution	54	21%
PCP: Outside Study Institution	165	65%
PCP: Missing Values	33	13%
**Total**	**252**	**100%**
Socioeconomic Risk: Low	152	60%
Socioeconomic Risk: Moderate	96	38%
Socioeconomic Risk: High	4	2%
Socioeconomic Risk: Missing Values	0	0%
**Total**	**252**	**100%**

**Table 2:** Summary data on study variables: Round 2 analysis (8–17 years).

	**N**	**%**
***ED Visit***		
One-Time ED Patient	96	69%
Repeat ED Patient	43	31%
**Total**	**139**	**100%**
***Individual Patient Demographics***		
Age: 8–12 years	46	33%
Age: 13–17 years	93	67%
Age: Missing Values	0	0%
**Total**	**139**	**100%**
Gender: Male	93	67%
Gender: Female	46	33%
Gender: Missing Values	0	0%
Total	139	100%
Race: Caucasian	7	5%
Race: African-American	123	88%
Race: Other	8	6%
Race: Missing Values	1	1%
**Total**	**139**	**100%**
Insurance: Medicaid	106	76%
Insurance: Private	32	23%
Insurance: Other	1	1%
Insurance: Missing Values	0	0%
**Total**	**139**	**100%**
***Individual Patient Risk Factors***		
Asthma Severity: Intermittent	6	4%
Asthma Severity: Mild-Persistent	39	28%
Asthma Severity: Moderate-Persistent	39	28%
Asthma Severity: Severe-Persistent	25	18%
Asthma Severity: Missing Values	30	22%
**Total**	**139**	**100%**
BMI: Normal	64	46%
BMI: Overweight	30	22%
BMI: Obese	30	22%
BMI: Missing Values	15	11%
**Total**	**139**	**100%**
Encounter Type: ED-to-inpatient	48	35%
Encounter Type: Transfer ED-to-inpatient	2	1%
Encounter Type: ED-only	89	64%
Encounter Type: Missing Values	0	0%
**Total**	**139**	**100%**
Encounter LOS: 0 days	78	56%
Encounter LOS: 1 day	40	29%
Encounter LOS: 2 days	14	10%
Encounter LOS: >2 days	7	5%
Encounter LOS: Missing Values	0	0%
**Total**	**139**	**100%**
PCP: Within Study Institution	33	24%
PCP: Outside Study Institution	88	63%
PCP: Missing Values	18	13%
**Total**	**139**	**100%**
Socioeconomic Risk: Low	85	61%
Socioeconomic Risk: Moderate	54	39%
Socioeconomic Risk: High	0	0%
Socioeconomic Risk: Missing Values	0	0%
**Total**	**139**	**100%**

**Table 3:** Contingency table analysis (Round 1 analysis: 0–17 years).

	One-Time ED	Repeat ED	Total	%	Fisher’s Exact Test *p*-Value
***Individual Patient Demographics***					
Age: <5 years	26	12	38	15%	0.0424 *
Age: 5–9 years	66	53	119	47%	
Age: >9 years	68	27	95	38%	
**Total**	**160**	**92**	**252**	**100%**	
Gender: Male	106	62	168	67%	0.8903
Gender: Female	54	30	84	33%	
**Total**	**160**	**92**	**252**	**100%**	
Race: Caucasian	17	6	23	9%	0.5061
Race: African-American	131	81	212	84%	
Race: Other	11	5	16	6%	
**Total**	**159**	**92**	**251**	**100%**	
Insurance: Medicaid	113	69	182	73%	0.0447 *
Insurance: Private	43	15	58	23%	
Insurance: Other	3	6	9	4%	
**Total**	**159**	**90**	**249**	**100%**	
***Individual Patient Risk Factors***					
Asthma Severity: Intermittent	25	16	41	22%	0.9963
Asthma Severity: Mild-Persistent	53	28	81	43%	
Asthma Severity: Moderate-Persistent	37	21	58	31%	
Asthma Severity: Severe-Persistent	5	3	8	4%	
**Total**	**120**	**68**	**188**	**100%**	
BMI: Normal	72	59	131	60%	0.1487
BMI: Overweight	32	14	46	21%	
BMI: Obese	28	14	42	19%	
**Total**	**132**	**87**	**219**	**100%**	
Encounter Type: ED-to-inpatient	56	35	91	36%	0.4005
Encounter Type: Transfer ED-to-inpatient	4	0	4	2%	
Encounter Type: ED-only	100	57	157	62%	
**Total**	**160**	**92**	**252**	**100%**	
Encounter LOS: 0 days	87	48	135	54%	0.2113
Encounter LOS: 1 day	49	29	78	31%	
Encounter LOS: 2 days	17	6	23	9%	
Encounter LOS: >2 days	6	9	15	6%	
**Total**	**159**	**92**	**251**	**100%**	
PCP: Within Study Institution	35	19	54	25%	0.5235
PCP: Outside Study Institution	98	67	165	75%	
**Total**	**133**	**86**	**219**	**100%**	
Socioeconomic Risk: Low	99	53	152	60%	0.0375 *
Socioeconomic Risk: Moderate	61	35	96	38%	
Socioeconomic Risk: High	0	4	4	2%	
**Total**	**160**	**92**	**252**	**100%**	

* Statistically significant at the 5% level.

**Table 4:** Logistic regression analysis (Round 1 analysis: 0–17 years).

**Dependent Variable: “One-Time ED” = 0, “Repeat ED” = 1** **N = 171 with Non-Missing Values**				
**Parameter**	**Category ¥**	**Coefficient Estimate**	***p*-Value**	**The Effect of Each Variable: Likelihood Ratio Test (*p*-Value)**
Asthma Severity	Mild-Persistent	−0.2223	0.4803	0.8853 (0.8290)
	Moderate-Persistent	−0.0882	0.7842	
	Severe-Persistent	0.1035	0.8617	
Age	5–9 years	0.6276	0.0159*	6.1365 (0.0465) *
	>9 years	−0.0718	0.8021	
Gender	Female	0.0701	0.7067	0.1413 (0.7070)
Race	African-American	0.4753	0.3013	2.3692 (0.3059)
	Other	0.4823	0.4503	
Insurance	Medicaid	−5.4012	0.9941	3.5896 (0.1662)
	Other	11.2819	0.9938	
BMI	Obese	0.0209	0.9472	2.0781 (0.3538)
	Overweight	−0.3514	0.283	
Socioeconomic Risk	High	11.5218	0.9937	3.7347 (0.1545)
	Moderate	−5.7338	0.9937	
**Odds Ratio for Each Age Group (*For Odds of Repeat Vs One-Time ED Visit*)**				
**Levels**	**Odds Ratio**	***p*-Value**	**CI**	
>9 years over 5–9 years	0.4968	0.0739	0.2306–1.0699	
>9 years over <5 years	1.6223	0.4386	0.4768–5.5187	
5–9 years over <5 years	3.2654	0.0444 *	1.0301–10.3518	

¥ The excluded category in each variable is the reference category. * Statistically significant at 5% level.

**Table 5:** Contingency table analysis (Round 2 analysis: 8–17 years).

	One-Time ED	Repeat ED	Total	%	Fisher’s Exact Test *p*-Value
***Individual Patient Demographics***					
Age: 8–12 years	64	29	93	67%	1.000
Age: 13–17 years	32	14	46	33%	
**Total**	**96**	**43**	**139**	**100%**	
Gender: Male	67	26	93	67%	0.3308
Gender: Female	29	17	46	33%	
**Total**	**96**	**43**	**139**	**100%**	
Race: Caucasian	5	0	5	4%	0.2228
Race: African-American	74	37	111	90%	
Race: Other	4	4	8	6%	
**Total**	**83**	**41**	**124**	**100%**	
Insurance: Medicaid	66	31	97	78%	0.6481
Insurance: Private	17	10	27	22%	
**Total**	**83**	**41**	**124**	**100%**	
***Individual Patient Risk Factors***					
Asthma Severity: Intermittent	16	9	25	23%	0.6947
Asthma Severity: Mild-Persistent	30	9	39	36%	
Asthma Severity: Moderate-Persistent	27	12	39	36%	
Asthma Severity: Severe-Persistent	4	2	6	6%	
**Total**	**77**	**32**	**109**	**100%**	
BMI: Normal	39	25	64	52%	0.3389
BMI: Overweight	22	8	30	24%	
BMI: Obese	22	8	30	24%	
**Total**	**83**	**41**	**124**	**100%**	
Encounter Type: ED-to-inpatient	39	11	50	36%	0.1255
Encounter Type: ED-only	57	32	89	64%	
**Total**	**96**	**43**	**139**	**100%**	
Encounter LOS: 0 day	49	29	78	56%	0.2959
Encounter LOS: 1 day	30	10	40	29%	
Encounter LOS: 2 days	12	2	14	10%	
Encounter LOS: >2 days	5	2	7	5%	
**Total**	**96**	**43**	**139**	**100%**	
PCP: Within Study Institution	23	10	33	27%	0.8305
PCP: Outside Study Institution	59	29	88	73%	
**Total**	**82**	**39**	**121**	**100%**	
Socioeconomic Risk: Low	56	29	85	61%	0.3502
Socioeconomic Risk: Moderate	40	14	54	39%	
**Total**	**96**	**43**	**139**	**100%**	

**Table 6:** Logistic regression analysis (Round 2 analysis: 8–17 years).

Dependent Variable: “One-Time ED” = 0, “Repeat ED” = 1 *N* = 102 with Non-Missing Values				
Parameter	Category ¥	Coefficient Estimate	*p*-Value	The Effect of Each Variable: Likelihood Ratio Test (*p*-Value)
Asthma Severity	Mild-Persistent	−0.3933	0.3334	1.0050 (0.8000)
	Moderate-Persistent	−0.0231	0.9539	
	Severe-Persistent	0.3149	0.6679	
Age	13–17 years	0.0495	0.8451	0.0381 (0.8451)
Gender	Female	0.0623	0.8079	0.0588 (0.8083)
Race	African-American	5.1649	0.9924	3.6171 (0.1639)
	Other	5.3758	0.9921	
Insurance	Medicaid	0.1295	0.6794	0.1735 (0.6769)
BMI	Obese	−0.2392	0.551	1.9616 (0.3750)
	Overweight	−0.1929	0.6021	
Socioeconomic Risk	Moderate	−0.3645	0.1451	2.1839 (0.1395)

¥ The excluded category in each variable is the reference category.

## Data Availability

The de-identified data analyzed in this study are available upon request from the corresponding author. The dataset contains patient-level data that could be identifiable by combining data elements. Therefore, the data are not publicly available due to concerns associated with data privacy & confidentiality.
